# Differences and Influencing Factors of Soil Bacterial Communities Under Different Forest Types on the Southern Slope of the Qilian Mountains

**DOI:** 10.3390/biology14080927

**Published:** 2025-07-23

**Authors:** Shuang Ji, Huichun Xie, Shaobo Du, Shaoxiong Zhang, Zhiqiang Dong, Hongye Li, Xunxun Qiu

**Affiliations:** 1 College of Geographical Sciences, Qinghai Normal University, Xining 810008, China; jishuang126@126.com (S.J.);; 2 Qilian Mountain Southern Slope Forest Ecosystem Research Station, Haidong 810500, China; 3 Key Laboratory of Medicinal Animal and Plant Resources of Qinghai-Tibetan Plateau, Xining 810008, China; 4 Team of Germplasm Resources Formation Mechanism and Utilization on the Qinghai-Tibetan Plateau, Xining 810008, China; 5 School of Life Sciences, Qinghai Normal University, Xining 810008, China; 6 Innovation and Intelligence Introduction Base for Plateau Resources Ecology and Sustainable Development, Xining 810008, China

**Keywords:** southern slope of the Qilian Mountains, forest type, soil bacterial diversity, influencing factors

## Abstract

Understanding the distribution patterns of soil bacterial community structure and diversity across different forest types is essential for elucidating the mechanisms underlying microbial community assembly and its ecological drivers, particularly under the pressures of climate change. In this study, we examined six forest types—including four monocultures and two mixed-species stands—to systematically evaluate the structural composition, diversity metrics, and functional potential of soil bacterial communities. Significant differences in microbial structure and functional composition were observed among forest types. Mixed forests exhibited higher soil nutrient levels, more complex structures, and greater water retention capacity, resulting in significantly higher bacterial and functional diversity compared to monoculture forests. Bacterial diversity was greater in subsurface layers than in surface layers. Surface communities in monoculture forests showed relatively high structural heterogeneity, whereas deeper communities in mixed forests displayed more pronounced differentiation. The dominant bacterial phyla were mainly related to carbon and nitrogen metabolism, compound degradation, and anaerobic photosynthesis. Surface bacterial communities were primarily influenced by catalase activity, alkali-hydrolysable nitrogen, bulk density, and pH, whereas subsurface communities were largely controlled by pH, with supplementary regulation by nitrogen and potassium availability. Therefore, forest type and soil depth jointly influence the diversity, composition, and functional attributes of soil microbial communities by modulating soil physicochemical conditions.

## Simple Summary

This study explored how different forest types influence the diversity and composition of soil bacterial communities in the Qilian Mountains. By comparing six forest types, including both single-species and mixed forests, researchers found that mixed forests had healthier soils, more nutrients, and better water retention, which supported more diverse and active bacterial communities. Deeper soil layers generally had higher bacterial diversity than surface layers. The type of forest and soil depth influenced bacterial communities by changing soil conditions such as pH, density, and nutrient levels. These findings enhance our understanding of forest ecosystem functioning and can inform more effective forest management strategies in response to climate change.

## 1. Introduction

In terrestrial ecosystems, forests occupy a pivotal position owing to their high complexity and multifunctionality, playing a crucial role in regulating global carbon (C) balance, conserving biodiversity, and maintaining climate stability [1]. Soil bacterial communities, the most active microbial groups in forest soil systems, play a central role in essential ecological processes such as organic matter decomposition and nutrient cycling. They are vital to sustaining soil functionality and ecological stability [2]. However, variations in forest vegetation types, driven by differences in species composition, litter characteristics, and root exudates, may induce substantial alterations in the composition and functional capacity of soil microbial communities [3]. At present, the regulatory mechanisms by which forest types influence soil bacterial communities are still insufficiently understood or explored, particularly under extreme ecological conditions such as those in alpine or plateau regions [4]. Therefore, investigating changes in soil bacterial communities across different forest types in cold alpine regions is of considerable scientific importance for elucidating the microbial mechanisms underpinning the responses of forest ecosystems to climate change and for promoting sustainable forest management. Soil bacteria represent the core driving force of material cycling and energy transformation in forest ecosystems [5], regulating key processes such as soil nutrient transformation and organic matter decomposition [6]. The physicochemical properties of plant litter and root exudates significantly vary across different forest ecosystem types, thereby exerting profound effects on the growth and metabolic activities of soil microorganisms and consequently shaping the structure and functional characteristics of microbial communities [7].

Litter, an important C source for soil microorganisms, varies in quality across vegetation types, which directly affects its decomposition rate and the composition of its byproducts. This, in turn, alters the soil microenvironment and ultimately leads to differentiation in bacterial community composition [8]. Root exudates, such as polysaccharides, organic acids, phenolic compounds, and extracellular enzymes selectively stimulate or inhibit specific bacterial taxa, thereby restructuring the soil bacterial community [9]. Furthermore, microbial communities associated with different plant species exhibit marked differences in their capacities for C and nitrogen (N) enrichment, which subsequently influences soil enzyme activities and availability of other soil nutrients [10]. These biochemical factors synergistically interact to drive the dynamic evolution of bacterial community structure and diversity. To date, numerous studies have examined soil bacterial community composition and diversity in various forest ecosystems. For instance, Thacker and Quideau [11] investigated the microbial communities in soils under Populus and Picea forests in western Canada. Their results showed that Populus has a higher influence than Picea on rhizosphere microbial composition and functional potential, thereby highlighting that the changes in aboveground vegetation have relatively high direct and profound effects on belowground microbial communities. Similarly, Hernández-Cáceres et al. [12], in their study of *Vaccinium myrtillus*, *Juniperus communis*, and *Picea abies* in the French Alps, found that soil physicochemical properties are the primary drivers of microbial activity, while microbial abundance, diversity, and functionality directly impact soil enzyme activities. Vegetation creates distinct microenvironments, primarily by altering the root chemical traits that mediate these relationships. These findings collectively underscore the significant impact of tree species on the structure and function of soil microbial communities, and specific soil enzyme activities. However, owing to variations in study regions and selected forest types, identifying the underlying drivers of microbial and enzymatic changes across forest types remains substantially limited. This is particularly true in alpine and high-latitude regions, where cold temperatures and oligotrophic conditions prevail and forest distribution is sparse. In such regions, the relationship between vegetation and microbial communities remains poorly understood [13]. Given that alpine forest ecosystems are ecologically fragile and highly sensitive to climate change, investigating soil bacterial communities and enzyme activities across different forest types in these environments is crucial to understanding microbial dynamics in alpine forest ecosystems and providing a scientific foundation for biodiversity conservation and ecosystem restoration under ongoing climate change.

The Qinghai–Tibet Plateau, characterized by its high elevation, intense solar radiation, hypoxic conditions, substantial variations in diurnal temperature, and marked spatial heterogeneity in hydrothermal distribution, has become a focal region for biodiversity research [14]. Situated in the northeastern margin of the Plateau, the Qilian Mountains play a pivotal role in the ecological security of arid northwestern China. The region exhibits significantly higher average altitudes than the surrounding areas, pronounced vertical relief, and sharp climatic gradients, which together shape typical alpine ecosystems. These conditions give rise to a complete and distinctive zonation of vertical vegetation, which supports a high level of biodiversity [15,16]. Such unique geographical and ecological characteristics make the Qilian Mountains a natural laboratory for investigating complex interactions among vegetation, environment, and microorganisms within alpine ecosystems. Previous studies on the soil microbial communities in the Qilian Mountains have primarily focused on grasslands [17], glacier permafrosts [18], and various land use types [19]. Few scholars have explored the soil microbes in different forest types of the Qilian Mountains, which has, to some extent, limited the understanding of the variations in soil microbial communities across different forest ecosystems in alpine regions.

In this study, we selected the Huzhu Beishan Forest Farm on the southern slope of the Qilian Mountains as our research site. We focused on four types of pure and two types of mixed forests for analyzing the diversity and functional composition of soil bacterial communities and the environmental factors influencing them. We aimed to elucidate the response patterns and primary drivers of soil bacterial communities under different forest types to provide foundational data for advancing our understanding of microbial ecological mechanisms in alpine forest ecosystems and for supporting future efforts in forest conservation and management in high-altitude regions.

## 2. Materials and Methods

### 2.1. Study Area and Experimental Design

The study was conducted on the southern slope of the Qilian Mountains, located in Qinghai Province, in the northeastern part of the Qinghai–Tibet Plateau. Geographically, the area extends from 98°08′13″ E to 102°38′16″ E, and 37°03′17″ N to 39°05′56″ N, covering an area of approximately 24,000 km^2^. The elevation ranges between 2286 and 5210 m, with an average elevation of 3800 m. The region experiences 2200–2900 h of annual sunshine and has a low mean annual temperature of approximately −5.9 °C. The plant-growing season coincides with relatively warm temperatures and periods of increased precipitation, with most rainfall concentrated between June and August. Annual precipitation averages between 300–400 mm. Winters are long and cold, whereas summers are short and cool, typical of alpine ecosystems. The terrain is predominantly mountainous, with substantial variation in elevation. The interaction of complex topography and climatic variability has resulted in a well-defined zonation of vertical vegetation, including grasslands, shrublands, and forests [20]. The forest ecosystems in this area are dominated by boreal coniferous species such as *Picea crassifolia*, *Picea wilsonii*, *Juniperus squamata*, and *Pinus tabuliformis*. Common broadleaf species include *Betula platyphylla*, *Betula albo-sinensis*, *Populus* spp., and other birch taxa. Major soil types in the study area include mountain forest and cinnamon soil, calcareous kastanozems, chernozems, alpine meadow, meadow, and alpine desert soil [21].

### 2.2. Sampling and Treatment

Between July and August 2023, soil sampling was conducted at the Huzhu Beishan Forest Farm located on the southern slope of the Qilian Mountains. Six forest types were selected, including four monoculture (*P. wilsonii*, *Betula*, *J. squamata*, and *P. tabuliformis*), and two mixed (conifer–broadleaf and broadleaf) forests (Table 1). For each forest type, three plots of 20 m × 20 m with similar site conditions and vegetation structure were established, totaling 18 plots. The distance between plots exceeded 100 m to ensure spatial independence. Within each plot, three 10-m^2^ subplots were designated. In each subplot, three random sampling locations were selected. At each sampling site, soils were collected from two depth intervals: 0–20 cm (designated as topsoil) and 20–40 cm (subsoil). For each layer, five cores were extracted along an S-pattern trajectory using a sterilized soil auger (Top Instrument, Hangzhou, China). The cores corresponding to the same depth were homogenized to form one representative composite sample [22]. In total, 108 composite samples were generated. Each of these was subsequently split into two portions: one fresh portion was reserved for soil enzyme activities were measured using commercial assay kits (Suzhou Comin Biotechnology Co., Ltd., Suzhou, China), while the other was air-dried for physicochemical property measurements, with coarse debris and undecomposed plant residues removed. In addition, under strict sterile conditions (with all tools sterilized by autoclaving), microbial soil samples were collected following the same protocol. Composite samples from both depths were immediately stored in liquid nitrogen (Yipu Gas Co., Ltd., Xi’an, China) at −80 °C for subsequent microbial analysis. All soil samples were preserved for microbial DNA was extracted using the Soil Genomic DNA Extraction Kit (Tiangen Biotech, Beijing, China)

### 2.3. Soil Physicochemical Properties

The mechanical composition of the soil samples was assessed using a laser diffraction particle size analyzer (Mastersizer 2000, Malvern Instruments, Malvern, UK), in accordance with international soil classification standards [23,24]. Gravimetric water content (SWC) was determined through oven drying, and bulk density (BD) was measured using the core method with a ring knife method. Soil electrical conductivity (EC) and pH were measured using a conductivity meter (LEIMi, Shanghai Yi Electrical Scientific Instrument Co., Ltd., Shanghai, China) and a pH meter (Beijing Sartorius Scientific Instrument Co., Ltd., Beijing, China), respectively. Total carbon (TC), soil organic carbon (SOC), and total nitrogen (TN) concentrations were quantified with an elemental analyzer (FlashSmart, Thermo Fisher Scientific, Waltham, MA, USA). The contents of total phosphorus (TP) and available phosphorus (AP) were evaluated using the antimony–molybdenum blue colorimetric method (Shimadzu, Kyoto, Japan). NaOH fusion followed by flame photometry (Shanghai Yidian Analytical Instrument Co., Ltd., Shanghai, China) was employed to determine total potassium (TK) and available potassium (AK). Alkali-hydrolyzable nitrogen (AN) was analyzed through the alkali diffusion technique using instrumentation from Plander (Wertheim, Germany). Enzyme activities, including those of sucrase (SUC), urease (URE), alkaline phosphatase (ALP), and catalase (CAT), were determined according to the procedures outlined in the Soil Agrochemical Analysis manual [25].

### 2.4. Soil DNA Extraction, Amplification by Polymerase Chain Reaction (PCR), and Illumina Sequencing

Soil genomic DNA was extracted using the E.Z.N.A.^®^ Soil DNA Kit (Omega Bio-tek, Norcross, GA, USA), following the manufacturer’s standard protocol. DNA integrity was confirmed via 1% agarose gel electrophoresis, while its concentration and purity were determined with a NanoDrop 2000 spectrophotometer (Thermo Fisher Scientific, Waltham, MA, USA). The hypervariable V3–V4 region of the bacterial 16S rRNA gene was targeted for amplification using primers 338F (5′-ACTCCTACGGGAGGCAGCAG-3′) and 806R (5′-GGACTACHVGGGTWTCTAAT-3′) [26], each tagged with a unique sample-specific barcode. PCR products were visualized using 2% agarose gel electrophoresis and purified using a commercial PCR Clean-Up Kit (YuHua Biotech, Shanghai, China). Amplicon concentrations were quantified using a Qubit 4.0 fluorometer (Thermo Fisher Scientific, Waltham, MA, USA). Sequencing was performed using the Illumina PE300 or PE250 platform at Majorbio Bio-Pharm Technology Co., Ltd. (Shanghai, China).

### 2.5. Statistical Analysis

Alpha diversity indices were calculated using the Mothur software platform (v1.48.0; http://www.mothur.org/wiki/Calculators, accessed on 20 July 2025). Statistical differences in alpha diversity among groups were evaluated using the Wilcoxon rank-sum test. To examine variations in bacterial community composition, Bray–Curtis dissimilarity matrices were employed. To assess associations between bacterial community composition and soil physicochemical properties, redundancy analysis (RDA) was conducted. Linear regression analysis was performed to evaluate the relationships between key soil physicochemical variables (identified in the RDA) and bacterial alpha diversity indices. Statistical analyses were conducted using IBM SPSS Statistics (version 26, IBM Corp., Armonk, NY, USA) and R software (version 4.0.0). Analysis of variance (ANOVA) and Duncan’s test were used to determine whether differences in the same index among different forest types within the same soil layer were significant. Meanwhile, paired-sample *t*-tests were applied to assess differences in the same index between two deep soil layers within the same forest type (*p* < 0.05).

## 3. Results

### 3.1. Soil Characteristics Under Different Forest Types

#### 3.1.1. Soil Physicochemical Properties Across Forest Types

**Figure 1:**
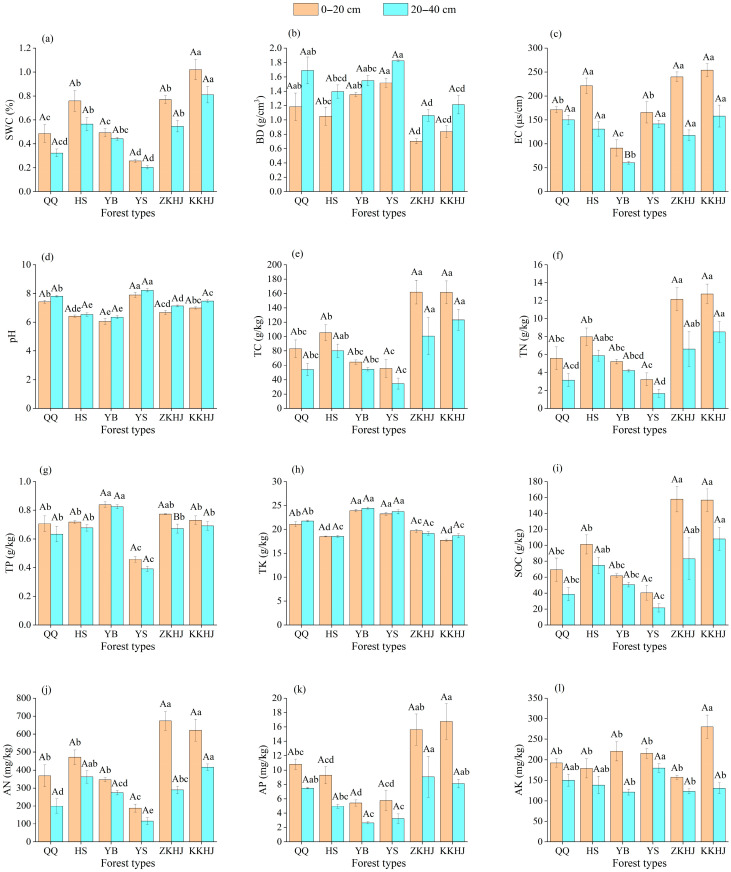
Soil physicochemical properties under different forest types and soil depths (n = 9). (**a**) SWC: soil water content; (**b**) BD: bulk density; (**c**) EC: electrical conductivity; (**d**) pH; (**e**) TC: total carbon; (**f**) TN: total nitrogen; (**g**) TP: total phosphorus; (**h**) TK: total potassium; (**i**) SOC: soil organic carbon. (**j**) AN: alkali-hydrolyzable nitrogen; (**k**) AP: available phosphorus; (**l**) AK: available potassium. Uppercase letters above the bars indicate significant differences in soil physicochemical properties between different soil depths within the same forest type, while lowercase letters indicate differences between different forest types at the same soil depth (*p* < 0.05). Bars sharing the same letter are not significantly different (*p* > 0.05). The error bars show the standard error of the dataset. Variables shown: QQ: pure forest of *Picea wilsonii*; HS: pure forest of *Betula*; YB: pure forest of *Juniperus squamata*; YS: pure forest of *Pinus tabuliformis*; ZKHJ: mixed coniferous and broadleaved forest; KKHJ: mixed broadleaved forest.

Notable disparities in soil physicochemical attributes were observed among the six forest ecosystems at both surface (0–20 cm) and subsurface (20–40 cm) depths (Figure 1). The broadleaf–conifer mixed stand (KKHJ) and the broadleaf-dominated mixed forest (ZKHJ) exhibited relatively higher soil moisture levels compared to the monoculture plantations (Figure 1a). Specifically, the KKHJ site recorded the maximum soil water content, surpassing all other forest types with statistical significance (*p* < 0.05). In contrast, the *Pinus tabuliformis* (YS) forest showed the lowest soil moisture values, significantly below those of the other five forest types (*p* < 0.05). These results suggest that mixed forests possess enhanced water-holding capacity, while pure stands, particularly YS, may be more susceptible to drought stress. The YS forest showed the highest soil BD, which was significantly higher than that of the *Betula* (HS), ZKHJ, and KKHJ forests (*p* < 0.05), suggesting that broadleaf forest soils have lower BD and more porous structures than those of coniferous stands (Figure 1b). Among all forest types, the *Picea* (YB) stand exhibited the minimum soil electrical conductivity (EC), with values significantly lower than those recorded in the other five forest ecosystems (*p* < 0.05), indicating relatively low levels of active mineral ions (Figure 1c). The observed soil pH in a studied region spanned a range from 6.05 to 8.23, with an average of 7.08, indicating slightly alkaline soils. The *Pinus tabuliformis* (YS) forest exhibited the highest soil pH, significantly exceeding those observed in the other five forest types (*p* < 0.05) (Figure 1d). In contrast, the mixed stands—broadleaf mixed (ZKHJ) and broadleaf–conifer mixed (KKHJ)—exhibited significantly higher concentrations of TC, TN, SOC, AN, AP, and AK, with the most pronounced enrichment observed in the 0–20 cm topsoil layer (Figure 1e,f,i–l). This suggests that mixed forest soils are more nutrient-rich and have higher nutrient supply capacities than monoculture forest soils. In contrast, the YS forest had the lowest values of TC, TN, TP, SOC, AN, and AK, indicating limited bioavailable C and N nutrients. The YB forest exhibited the highest TP levels, which were significantly higher than those in the KKHJ, *Pinus* (QQ), HS, and YS forests (*p* < 0.05), across both soil layers (Figure 1g). Among the six forest types, the highest TK content was observed in the YB forest. Moreover, TK content in the three coniferous forests (YB, YS, QQ) was markedly greater than the values observed in the three broadleaf and mixed forests (Figure 1h).

In terms of differences based on soil layers, the 0–20 cm topsoil across all six forest types exhibited higher values of SWC, EC, TC, TN, TP, SOC, AN, AP, and AK than did the 20–40 cm subsoil layer. Conversely, topsoil exhibited lower bulk density (BD) and pH compared to the subsoil layer. Most forest types showed a higher TK content in the 20–40 cm layer than in the 0–20 cm layer, except for the ZKHJ, where the TK content was relatively high in the topsoil. Overall, the variation trends of soil physical and chemical properties between the two soil layers were generally consistent across the six forest types.

#### 3.1.2. Soil Particle Size Composition Across Forest Types

**Figure 2:**
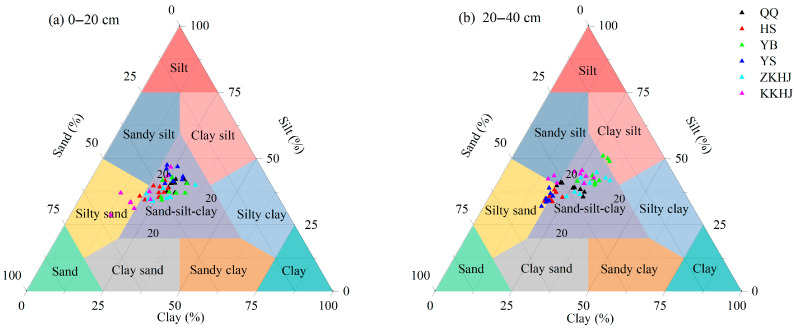
Soil particle size composition under different forest types and soil layers (n = 54). QQ: pure forest of *Picea wilsonii*; HS: pure forest of *Betula*; YB: pure forest of *Juniperus squamata*; YS: pure forest of *Pinus tabuliformis*; ZKHJ: mixed coniferous and broadleaved forest; KKHJ: mixed broadleaved forest. (**a**) 0–20 cm. (**b**) 20–40 cm.

Overall, the soils were dominated by silt particles (38.80%), followed by sand (36.13%), whereas the clay content was relatively low (25.08%) (Figure 2). Both the QQ and ZKHJ forests exhibited relatively compact and balanced particle size distributions in both soil layers, mainly clustered within the “sand–silt–clay” zone, indicating a well-structured soil texture. The HS forest had particle size distributions mainly between the “sand–silt–clay” and “silty sand” zones, with a slight shift toward sand compared to the QQ forest, suggesting a relatively loose soil structure. The YB forest showed relatively high proportions of silt and clay, and some subsoil (20–40 cm) samples fell into the “clay silt” zone, indicating a fine texture in deep soil layers. The YS forest had particle size distributions between the “sand–silt–clay” and “silty sand” zones, with relatively high sand content observed in the 20–40 cm soil layer. In contrast, the KKHJ forest exhibited a highly dispersed distribution, with samples spanning multiple texture zones including “sand–silt–clay”, “sandy silt”, and “clay silt”, indicating a relatively complex soil physical structure in the understory.

#### 3.1.3. Soil Enzyme Activities Across Vegetation Types

**Figure 3:**
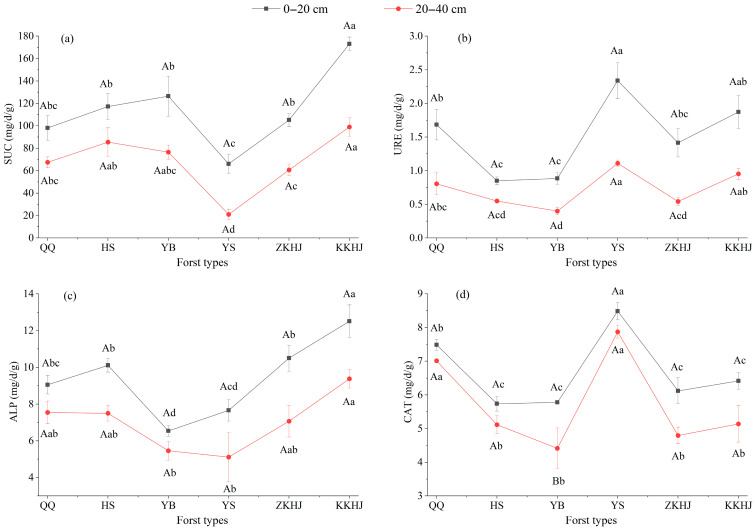
Soil enzyme activities under different forest types and soil depths (n = 9). (**a**) SUC: sucrase; (**b**) URE: urease; (**c**) ALP: alkaline phosphatase; (**d**) CAT: catalase. Uppercase letters indicate significant differences in soil physicochemical properties between different soil depths within the same forest type, while lowercase letters represent differences between different forest types at the same soil depth (*p* < 0.05). Bars sharing the same letter indicate no significant differences (*p* > 0.05). The error bars show the standard error of the mean. QQ: pure forest of *Picea wilsonii*; HS: pure forest of *Betula*; YB: pure forest of *Juniperus squamata*; YS: pure forest of *Pinus tabuliformis*; ZKHJ: mixed coniferous and broadleaved forest; KKHJ: mixed broadleaved forest.

Soil enzyme activities significantly varied with forest type and soil depth (Figure 3). The KKHJ forest exhibited the highest activities of SUC and ALP in both soil layers. Notably, ALP and SUC activities in the 20–40 cm layer were significantly higher in the KKHJ forest than in the other five forest types (*p* < 0.05), suggesting that broadleaf mixed forests have markedly higher C and phosphorus (P) transformation potentials than monoculture forests. In contrast, the YS forest exhibited the lowest SUC activity (*p* < 0.05), indicating limited C transformation capacity. The YB forest had significantly lower ALP activity in the 20–40 cm layer than that of QQ, HS, ZKHJ, and KKHJ forests (*p* < 0.05), reflecting relatively weak phosphorus mobilization in the subsoil. Interestingly, the YS forest displayed the highest activities of URE and CAT across both soil layers. URE activity in the YS forest was significantly higher than that in the QQ, HS, YB, and ZKHJ forests across both soil layers. In the 0–20 cm topsoil layer, CAT activity in the YS forest was significantly higher than that observed in the HS, YB, ZKHJ, and KKHJ forests. In the 20–40 cm subsoil layer, CAT activity was significantly greater than that observed in all five of the other forest types (*p* < 0.05). These results suggest that the YS forest has a highly active rhizosphere N metabolism and excellent capacity to buffer oxidative stress. In contrast, the HS and YB forests exhibited relatively low URE and CAT activities, indicating relatively weak N activation and antioxidant enzymatic systems.

### 3.2. Composition of Soil Bacterial Communities Across Vegetation Types

**Figure 4:**
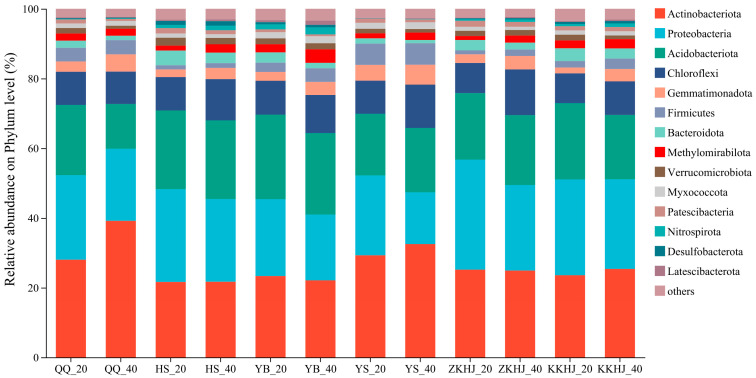
Relative abundance of soil bacterial communities under different forest types and soil layers (n = 9). The suffixes _20 and _40 refer to the 0–20 cm and 20–40 cm soil layers, respectively. QQ: pure forest of *Picea wilsonii*; HS: pure forest of *Betula*; YB: pure forest of *Juniperus squamata*; YS: pure forest of *Pinus tabuliformis*; ZKHJ: mixed coniferous and broadleaved forest; KKHJ: mixed broadleaved forest.

Fourteen dominant bacterial phyla, each with a relative abundance exceeding 1%, were detected across the six forest types (Figure 4). These included *Actinobacteriota*, *Proteobacteria*, *Acidobacteriota*, *Chloroflexi*, *Gemmatimonadota*, *Firmicutes*, *Bacteroidota*, *Methylomirabilota*, *Verrucomicrobiota*, *Myxococcota*, *Patescibacteria*, *Nitrospirota*, *Desulfobacterota*, and *Latescibacterota*. Among them, *Acidobacteriota*, *Proteobacteria*, *Actinobacteriota*, and *Chloroflexi* were dominant across all forest types and soil layers, collectively accounting for more than 60% of the total relative abundance. In both the QQ and YS forests, a greater relative abundance of *Acidobacteriota* was observed in the subsoil layer (20–40 cm) compared to the surface soil (0–20 cm). Conversely, *Proteobacteria* were more prevalent in the upper soil layer (0–20 cm) across all six forest types. Although the relative abundances of *Actinobacteriota* and *Chloroflexi* differed with forest type and soil depth, these variations were not statistically significant.

**Figure 5:**
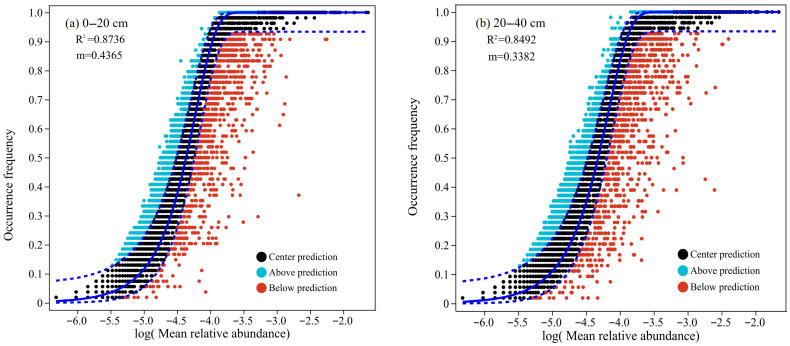
The bacterial neutral community model (NCM) under different forest types and soil layers (n = 54). (**a**) 0–20 cm. (**b**) 20–40 cm.

The results of neutral community model (NCM) fitting (Figure 5) showed a strong explanatory power for bacterial community structure. For the 0–20 cm topsoil, the model fit was R^2^ = 0.87 with a migration rate (m) of 0.44, while for the 20–40 cm subsoil R^2^ = 0.85 and m = 0.34. These results suggest that the neutral model explained more than 85% of the variation in the bacterial community structure at both depths, indicating that stochastic processes played a dominant role. However, the low m suggested limited bacterial dispersal or migration across communities. The topsoil exhibited higher model fit and m than the subsoil, implying that microbial communities in the 0–20 cm layer were relatively strongly influenced by neutral processes such as random dispersal and ecological drift. The increased density of red points in the 20–40 cm model indicated an enhanced influence of niche-based (deterministic) processes. Taken together with the relatively stable bacterial community composition across forest types and minimal fluctuations in the abundance of dominant taxa, these results suggest that soil bacterial communities in the study area closely aligned with the assumptions of the neutral model, in which stochastic processes dominate community assembly and species dispersal is constrained and deterministic environmental filtering plays a comparatively minor role.

### 3.3. Alpha Diversity of Soil Bacterial Communities Across Vegetation Types

**Figure 6:**
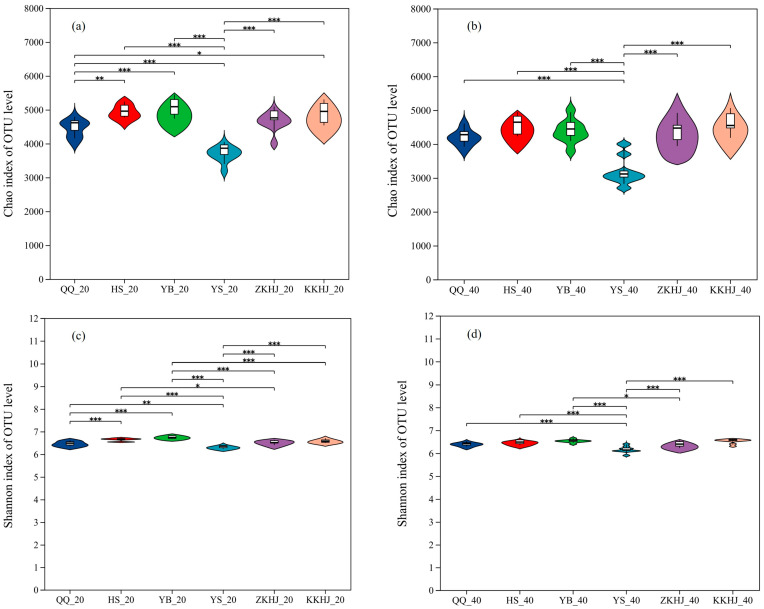
Variation in soil bacterial alpha diversity across forest types and soil layers (n = 9). (**a**) Chao index of soil bacteria in the 0–20 cm soil layer; (**b**) Chao index of soil bacteria in the 20–40 cm soil layer; (**c**) Shannon index of soil bacteria in the 0–20 cm soil layer; (**d**) Shannon index of soil bacteria in the 20–40 cm soil layer. The suffixes _20 and _40 indicate the 0–20 cm and 20–40 cm soil layers, respectively. The error bars represent the standard error of the data. Asterisks indicate significant differences between forest types for the same soil layer and parameter: * 0.01 < *p* ≤ 0.05, ** 0.001 < *p* ≤ 0.01, *** *p* ≤ 0.001. QQ: pure forest of *Picea wilsonii*; HS: pure forest of *Betula*; YB: pure forest of *Juniperus squamata*; YS: pure forest of *Pinus tabuliformis*; ZKHJ: mixed coniferous and broadleaved forest; KKHJ: mixed broadleaved forest.

The alpha diversity of soil bacterial communities was significantly affected by forest type, though the extent of impact varied among forest types (Figure 6). Among all forest types and soil layers, the *Pinus tabuliformis* (YS) stand displayed the lowest values on both the Chao and Shannon indices, with significantly reduced values compared to the other five forest types (*p* < 0.001 and *p* < 0.01, respectively). These results reflect reduced species richness and overall microbial diversity in the YS forest. In contrast, the YB, HS, and KKHJ forests showed relatively high Chao and Shannon indices, suggesting relatively rich and diverse microbial communities. The QQ and ZKHJ forests had moderate levels of species richness and diversity. Across all forest types, the 20–40 cm subsoil layer consistently exhibited Chao and Shannon indices higher than those of the 0–20 cm topsoil, suggesting that the deeper soil layer played a prominent role in influencing bacterial diversity within forest soil ecosystems.

**Figure 7:**
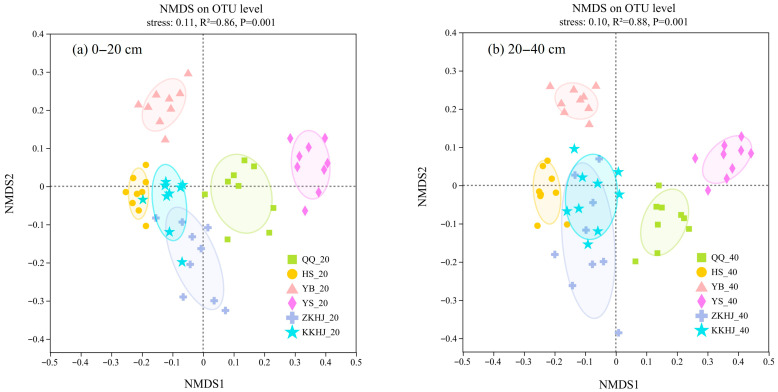
NMDS ordination depicting differences in soil bacterial community composition across forest types and soil layers (n = 9). The suffixes _20 and _40 refer to the 0–20 cm and 20–40 cm soil layers, respectively. QQ: pure forest of *Picea wilsonii*; HS: pure forest of *Betula*; YB: pure forest of *Juniperus squamata*; YS: pure forest of *Pinus tabuliformis*; ZKHJ: mixed coniferous and broadleaved forest; KKHJ: mixed broadleaved forest. (**a**) 0–20 cm. (**b**) 20–40 cm.

Non-metric multidimensional scaling (NMDS) based on operational taxonomic unit (OTU)-level data (Figure 7) showed that sample points from different forest types formed tight clusters within groups but were clearly separated between groups, suggesting that forest type exerted a significant influence on bacterial community composition in both topsoil and subsoil layers (*p* < 0.001). Overall, the microbial communities in the YB and YS forests were most distinctly separated from those in the other four forest types, suggesting highly specific microbial structures. In contrast, the communities in the ZKHJ and KKHJ forests partially overlapped, indicating the presence of similar microhabitats or microbial compositions. The QQ and HS forests showed relatively distinct clustering patterns, reflecting unique community structures in these forests. The 0–20 cm topsoil layer in the four monoculture forests (QQ, HS, YB, and YS) exhibited a higher dispersion of sample points than their 20–40 cm subsoil counterparts, indicating relatively high heterogeneity in the surface bacterial community structure. In contrast, the mixed forests (ZKHJ and KKHJ) showed the opposite trend, with relatively deep soil layers displaying highly dispersed community patterns.

### 3.4. Functional Diversity of Soil Bacterial Communities Across Vegetation Types

**Figure 8:**
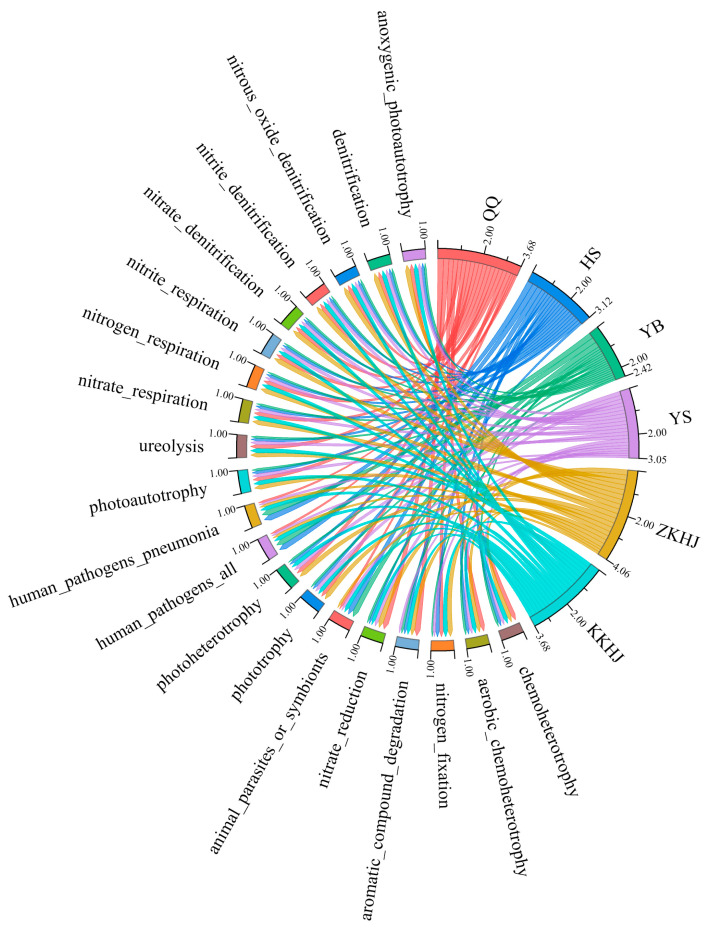
Predicted bacterial functional profiles predicted by FAPROTAX across different forest types (n = 18). QQ: pure forest of *Picea wilsonii*; HS: pure forest of *Betula*; YB: pure forest of *Juniperus squamata*; YS: pure forest of *Pinus tabuliformis*; ZKHJ: mixed coniferous and broadleaved forest; KKHJ: mixed broadleaved forest.

Using 16S rRNA gene-based taxonomic classification combined with functional annotation through the FAPROTAX database, a total of 63 putative functional groups were identified across soil bacterial communities from the six forest types. The 20 most abundant functions within each forest type were selected for visualization via chord diagrams (Figure 8), which highlighted distinct differences in functional composition between the forest ecosystems. These functions primarily clustered into three categories: (1) C and N metabolism (e.g., *chemoheterotrophy* and *nitrogen fixation*), (2) compound degradation (e.g., *aromatic compound degradation*), and (3) other specialized metabolic processes (e.g., *anoxygenic photoautotrophy*). Among the forest types, QQ and the two mixed forests (ZKHJ and KKHJ) exhibited relatively high bacterial functional diversity and redundancy, encompassing a broad range of ecological processes such as N cycling, organic matter decomposition, and C fixation. In contrast, the HS and YB forests showed relatively high functional representation associated with human pathogenicity, suggesting potential ecological risk and the need for further attention. The YS forest displayed relatively narrow functional diversity, with functions primarily related to N metabolism, indicating a highly functionally specialized microbial community. Overall, these findings suggest that forest types significantly shape the composition of soil bacterial functional groups by modifying microhabitat conditions, thereby influencing the functional capacity of forest ecosystems.

### 3.5. Key Environmental Factors Influencing Soil Bacterial Community Composition and Diversity Across Vegetation Types

**Figure 9:**
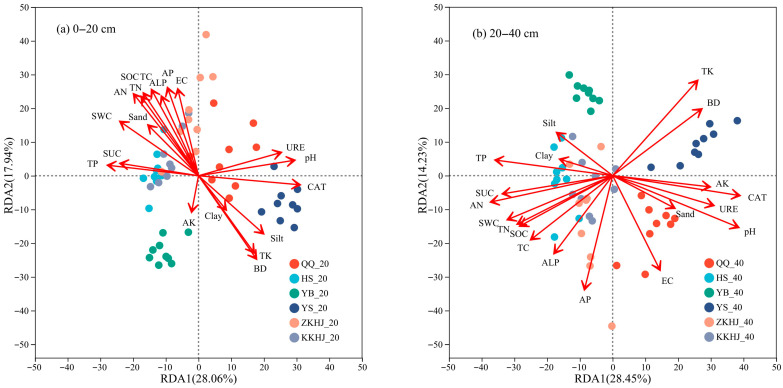
Redundancy analysis (RDA) of soil physicochemical properties, enzyme activities, and bacterial community structure across different forest types and soil layers (n = 9). The suffixes _20 and _40 refer to the 0–20 cm and 20–40 cm soil layers, respectively. QQ: pure forest of *Picea wilsonii*; HS: pure forest of *Betula*; YB: pure forest of *Juniperus squamata*; YS: pure forest of *Pinus tabuliformis*; ZKHJ: mixed coniferous and broadleaved forest; KKHJ: mixed broadleaved forest. (**a**) 0–20 cm. (**b**) 20–40 cm.

Figure 9 illustrates the relationships between soil physicochemical properties, enzyme activities, and bacterial community composition. In the 0–20 cm topsoil, the first and second axes of redundancy analysis (RDA) explained 28.06% and 17.94% of the total variance, respectively. Among the influencing variables, CAT activity emerged as the primary driver of bacterial community structure across forest types, followed by AN and BD. In the 20–40 cm subsoil, the first and second RDA axes accounted for 28.45% and 14.23% of the total variation, respectively, with soil pH also contributing to community differentiation. pH was the primary factor shaping bacterial communities in subsurface soils, followed by CAT, AN, and TK. The HS and KKHJ forests were strongly influenced by TP, SUC, and SWC. The YS and ZKHJ forests were strongly affected by CAT, pH, URE, and silt content. The YB forest was primarily influenced by TK, while the QQ forest was mainly associated with EC. These results indicate that different soil layers and forest types are shaped by distinct combinations of biotic and abiotic drivers, with enzyme activities and soil chemical parameters playing dominant roles.

## 4. Discussion

### 4.1. Effect of Forest Types on Soil Microbial Communities

Vegetation type can modulate the abundance and diversity of soil bacterial taxa via root-associated mechanisms, selectively fostering microbial communities that are beneficial to plant performance [27]. In the present study, we detected 14 dominant bacterial phyla (each with a relative abundance > 1%) across the six forest ecosystems. Among these, *Acidobacteriota*, *Proteobacteria*, *Actinobacteriota*, and *Chloroflexi* were the most prevalent—patterns that align with earlier observations reported on the southern slopes of the Qilian Mountains [27]. This pattern is likely a result of intense environmental filtering driven by the region’s severe alpine conditions, characterized by low temperatures, intense ultraviolet radiation, and arid climate. Only those microbial taxa adapted to such extreme conditions can survive. For example, *Actinobacteriota* and *Chloroflexi* possess cell-wall structures that confer resistance to drought and low temperature; *Acidobacteriota* dominates in oligotrophic environments, and *Proteobacteria* exhibits broad ecological versatility, thriving under various environmental conditions [28]. Moreover, a positive feedback relationship exists between dominant bacterial phyla and the dynamics of forest soil nutrients. These dominant groups play essential roles in soil nutrient cycling. For instance, *Acidobacteriota* is highly tolerant of environmental stress and can regulate soil pH, contributing to the maintenance of a neutral to slightly acidic soil environment in forests [29,30,31,32]. *Proteobacteria* is a key player in the N cycle, participating in processes such as N fixation, denitrification, and intermediate stages of nitrification, thereby enhancing N availability in forest soils [33,34,35,36]. *Actinobacteriota* can decompose complex organic matter, including cellulose and lignin, and transform it into forms that are accessible to plants. In the forests of the southern Qilian Mountains, abundant plant litter and root exudates provide ample substrates for actinobacteria, supporting their proliferation. *Chloroflexi* is functionally important in soil C and N cycling and contributes to maintaining forest soil fertility and ecological balance. Its ability to utilize diverse C and N sources allows its adaptation to various microhabitats found across different forest types, whether in pure stands of *Picea crassifolia* or *Betula*, or in mixed conifer–broadleaf forests [37]. These ecological traits explain why *Acidobacteriota*, *Proteobacteria*, *Actinobacteriota*, and *Chloroflexi* have become dominant phyla in the forest soils of the southern Qilian Mountains.

Across all six forest types, in all cases, the relative abundance of Proteobacteria was consistently greater in the surface (0–20 cm) soil layer than in the subsoil (20–40 cm). This pattern aligns with observations reported in earlier studies [38,39], and is largely driven by variations in nutrient levels and oxygen concentrations across different soil depths. The surface soil (0–20 cm) is rich in litter and root exudates, which provide abundant C sources essential for microbial growth. Additionally, its higher porosity enhances oxygen diffusion, creating favorable conditions for aerobic bacteria, such as *Proteobacteria*, to thrive [40]. In contrast, in both the QQ and YS forests, *Acidobacteriota* showed a markedly higher relative abundance in the 20–40 cm subsoil layer compared to the topsoil. This aligns with the ecological strategy of *Acidobacteriota*, which prefers low-C and nutrient-poor environments. Our results also support this, as the 20–40 cm soils in the QQ and YS forests had relatively low levels of TN, TC, SOC, TP, AN, AP, and AK. Moreover, the relatively high R^2^ values obtained from the neutral community model (NCM) suggest that stochastic mechanisms—including random dispersal, colonization, and extinction—predominantly influence the assembly of soil bacterial communities. However, the low value suggests that actual microbial dispersal events are limited. Notably, the topsoil microbial communities exhibited higher migration rates than did those in the subsoil. This pattern aligns with previous findings [41,42]. Forest soil porosity and aggregate structure can limit microbial movement, while the surface soil that is enriched in organic matter forms localized microhabitats or “micro-niches” that reduce the ecological drive for downward dispersal. Additionally, the SWC observed in the subsoil across all forest types was relatively low, indicating limited moisture dynamics, which further restricts the vertical dispersal of soil microbial communities.

### 4.2. Effect of Forest Type on Soil Microbial Diversity

Forest type exerted a significant influence on the diversity of soil bacterial communities. The YS forest had the lowest species richness and diversity among the six forest types, while the YB, HS, and KKHJ forests exhibited relatively high richness and diversity. The QQ and ZKHJ forests were intermediate. These differences are strongly associated with variations in soil physicochemical properties among forest types. Previous studies have confirmed this linkage. For example, bacterial diversity across different forest types (including coniferous, broadleaf, and mixed forests) is closely correlated with soil physicochemical conditions, particularly showing a negative correlation with soil pH and a strong dependency on SOC and TN contents [43]. Our results support these findings. Among the six forest types, YS had lower concentrations of SWC, TC, TN, TP, SOC, and AN, but higher pH and BD than the others.

The relatively low bacterial richness and diversity observed in YS may be attributed to several factors. The first is water limitation. *P. tabuliformis* may consume a large amount of soil water. As these trees age, soil moisture decreases, creating relatively dry conditions that are unfavorable for moisture-dependent bacterial taxa [44]. In contrast, species like *Juniperus* and *Picea* have relatively highly deep root systems and canopy structures that enhance rainfall interception and soil moisture retention, thereby providing highly suitable hydrological conditions for microbial communities. The second is low nutrient input and turnover. Litter decomposition in *P. tabuliformis* forests is relatively slow, resulting in limited nutrient return to the soil. Additionally, its root system may have relatively low nutrient uptake efficiency, contributing to reduced concentrations of bioavailable C, N, and P for microbial metabolism [45,46]. In contrast, broadleaf trees, such as *Betula*, produce a relatively high amount of bark and litter, which decompose more rapidly, thereby increasing soil porosity and aeration, and improving the microenvironment for bacterial colonization.

Regarding soil depth, the 20–40 cm subsoil generally exhibited higher Chao and Shannon indices than did the 0–20 cm topsoil, suggesting that subsurface environments exert relatively a strong influence on bacterial diversity. This observation is consistent with a previous finding [47]. The subsoil has relatively stable temperature, moisture, and pH conditions and experiences relatively low anthropogenic disturbance. Such environments are conducive to the long-term persistence of K-strategist microbial taxa. Although subsoil nutrient concentrations (e.g., TN, TP, AK, etc.) are generally lower than those in the surface layer (Figure 1), the relatively deep layer may offer unique ecological niches, such as low-oxygen microzones and mineral-associated organic matter sites, which selectively attract specialized bacterial communities [42]. Additionally, limited microbial dispersal and migration in relatively deep soil layers can lead to the formation of distinct and localized bacterial populations, resulting in the accumulation of distinct bacterial diversity over time [48]. This is further supported by the results of our NCM analysis.

The NMDS analysis at the OTU level showed that soil bacterial communities in the YB and YS forests were most clearly separated from those in the other four forest types, indicating a high degree of microbial structure specificity. In contrast, bacterial communities in the ZKHJ and KKHJ forests exhibited partial overlap, suggesting shared microhabitats or similar microbial compositions. The QQ and HS forests formed relatively distinct clusters, reflecting unique community structures. These patterns of microbial spatial segregation and overlap are primarily shaped by differences in microhabitats driven by forest type and complex plant–soil–microbe interactions [49,50,51,52,53]. The distinct separation of YB and YS from the other forest types may be attributed to specific vegetation traits; e.g., *P. tabuliformis* produces chemically simple litter with limited nutrient inputs, while *J. squamata* often releases aromatic secondary metabolites, both of which strongly affect microbial assemblages and lead to unique community compositions [54]. The observed overlap between ZKHJ and KKHJ is probably owing to similar species composition in these mixed forests, which promotes niche sharing and creates comparable soil physicochemical conditions, resulting in convergent microbial community structures [55]. For the QQ and HS forests, the distinct clustering likely reflects independent vegetation types and long-term successional trajectories of these forest stands, each fostering distinct microbial assemblages. The 0–20 cm topsoil in the four monoculture forests exhibited higher dispersion in bacterial communities compared to their 20–40 cm subsoil counterparts, indicating relatively high surface heterogeneity. In contrast, the mixed forests showed the opposite trend, with relatively deep soil communities displaying relatively high variability. This pattern can be explained by the simplified vegetation structure in monocultures, where the surface soil is more exposed to external environmental fluctuations (e.g., light, temperature, and moisture) and is affected by the decomposition rate and chemical composition of uniform litter, all of which strongly influence microbial spatial distribution [56]. Moreover, in monoculture forests, root systems are typically concentrated near the surface. The localized release of root exudates creates micro-scale nutrient gradients that selectively influence different microbial groups. Surface soil in monocultures also experiences relatively frequent activities by soil fauna, such as burrowing and feeding, which disrupt microbial uniformity and increase spatial heterogeneity [57]. In contrast, mixed forests exhibit more diverse root exudates across species that interact in relatively deep soil layers, leading to complex microbial microenvironments. The relatively high diversity of litter in these systems contributes to highly rich humus formation, which improves the structure of deep soil and enhances its buffering capacity against environmental fluctuations. This promotes niche differentiation among microbial taxa and increases the spatial heterogeneity of the microbial community [58].

The functional diversity of soil bacterial communities across the six forest types was mainly categorized into three types: C and N metabolism, compound degradation, and specialized metabolic processes such as anoxygenic photoautotrophy. Forest soils contain abundant organic matter, including plant litter and root exudates, which are rich in C and N. Soil bacteria participate in the decomposition and transformation of these materials through C and N metabolic pathways, thereby contributing to energy acquisition and nutrient turnover, while also playing critical roles in broader C and N cycling within ecosystems [59]. In addition, anoxygenic photoautotrophic bacteria can be found in specific forest soil niches, such as deep or anaerobic layers, utilizing light energy to fix carbon dioxide and water into energy-rich organic molecules, producing other oxidized products in the process. This form of metabolism enables bacteria to survive and proliferate in low-oxygen environments, while also supplying the ecosystem with additional organic matter and energy [39,60,61]. Mixed forests exhibit relatively high functional diversity and redundancy owing to higher heterogeneity in litter composition (e.g., C: N ratio and phenolic compounds) and diverse root exudates resulting from species mixtures [62]. In contrast, the QQ forest may accumulate more recalcitrant organic matter owing to the relatively slow decomposition of coniferous litter. This accumulation in turn may select for bacterial taxa with diverse degradation pathways, including lignin-degrading enzyme systems, which enhances functional diversity [54]. These ecological mechanisms support our findings that the QQ, ZKHJ, and KKHJ forest types displayed relatively high bacterial functional diversity and redundancy. Interestingly, the HS and YB forests exhibited higher relative abundances of functions associated with human pathogens, raising concerns about potential ecological risks. *Betula* litter is rich in water-soluble C and N and decomposes rapidly, which may create transient eutrophic microenvironments that promote the proliferation of opportunistic pathogens such as *Enterobacteriaceae*. In the case of *J. squamata*, the litter contains terpenoid antimicrobial compounds that may suppress beneficial bacteria (e.g., *Actinobacteria*) but allow resistant pathogens (e.g., *Pseudomonas aeruginosa*) to adapt and be enriched via specialized metabolic pathways [36]. Furthermore, both HS and YB forests had relatively low soil pH, which may favor acid-tolerant pathogens. By contrast, the bacterial community in the YS forest exhibited a relatively narrow functional profile, dominated by N-related pathways. This is probably because *Pinus* litter contains high levels of tannins and lignin, which decompose slowly, resulting in C inputs in recalcitrant forms. Consequently, microbial communities in YS soils may be forced to rely heavily on N-based metabolism, leading to a more functionally specialized structure [63].

### 4.3. Environmental Factors Jointly Regulate the Variation in Soil Microbial Communities Across Forest Types

The classical ecological theory of microbial community assembly suggests that environmental factors play a deterministic role in shaping soil microbial composition [64]. In the present study, different environmental drivers acted at varying depths. In the 0–20 cm topsoil, which is typically a zone of heightened microbial activity, CAT activity, N availability, soil BD, and pH emerged as the dominant factors influencing bacterial diversity. In contrast, the 20–40 cm subsoil, characterized by relatively stable environmental conditions, was primarily regulated by pH, with additional influence from enzyme activities as well as N and K contents. These findings align with those previously reported [28,59]. Across forest types, active root exudation, litter decomposition, and microbial respiration processes result in the production of reactive oxygen species, such as hydrogen peroxide, which can be neutralized by CAT, helping maintain redox balance and creating a favorable environment for bacterial survival and helping maintain redox balance. Soil N and K serve as crucial indicators of nutrient availability and energy supply for microorganisms. At the same time, shifts in microbial communities are closely tied to nutrient dynamics [65]. For instance, AN availability can directly influence microbial access to N resources, thereby either promoting or constraining bacterial growth and reproduction and ultimately affecting community richness and structure [66]. Soil bulk density and pH reflect the compaction level, pore structure, and acidity–alkalinity balance of the soil, physical and chemical thresholds that can directly constrain microbial habitat conditions and modulate microbial functions [28]. Soil pH, in particular, directly regulates enzyme activities, which in turn influence bacterial and fungal community composition [67], and also affects ion exchange capacity, indirectly altering nutrient availability [68]. Therefore, pH is closely intertwined with the availability of major nutrients, such as C, N, P, and K, and acts as a foundational factor controlling elemental biogeochemical cycles, with profound impacts on microbial diversity and community assembly. Additionally, BD is closely related to soil porosity, which governs both nutrient retention and oxygen diffusion in the soil matrix. Limited oxygen diffusion under the high BD conditions may restrict microbial proliferation, particularly for aerobic taxa, and consequently reduce overall microbial diversity [69].

Finally, although the precise mechanisms underlying the interactions between vegetation and soil microbial communities remain incompletely understood, vegetation type plays a pivotal role in shaping microbial communities, primarily through its influence on litter quality, root exudation, and the allocation of photosynthates, which collectively regulate material and energy fluxes within forest ecosystems. These processes additionally influence surface soil microclimatic conditions and nutrient cycling dynamics, ultimately shaping both the diversity and functional potential of soil microbial assemblages [70,71]. It is important to emphasize that this study was conducted under the natural, undisturbed conditions associated with the various forest types in this region. Nevertheless, factors like soil heterogeneity and anthropogenic disturbances may also shape microbial community structure and diversity. Future research should comprehensively account for all potential influencing factors to better support the management of alpine forest ecosystems.

## 5. Conclusions

Soil microbial community composition and diversity are jointly regulated by soil properties, nutrient availability, and vegetation characteristics, emphasizing the complex and integrated nature of plant–soil–microbe interactions. The six forest types on the southern slope of the Qilian Mountains exhibit significant ecological function differences at various soil depths. In mixed forests, soils at different depths exhibit higher nutrient availability, more complex soil structure, and stronger water retention capacity. These advantages are closely related to the rich plant species and the diversity of root systems in mixed forests, as well as their higher organic matter accumulation and the richness of soil microbial communities. The diverse vegetation in mixed forests regulates the soil’s physical and chemical properties, providing a more stable and varied environment for soil microorganisms, thereby promoting the potential for C and P transformations in the soil. These enzyme-mediated processes are crucial for maintaining soil ecological functions and facilitating nutrient cycling. In contrast, single-species forests, especially those of *Pinus tabuliformis*, although possessing a simpler vegetation structure, still display unique ecological functions under specific ecological conditions. The soil in pine forests tends to be more compacted, with a limited accumulation of organic matter, weak water retention capacity, and limited nutrient supply. This phenomenon is primarily attributed to the uniformity of root types in single-species forests and the lower nutrient availability in the soil. However, the uniqueness of pine forests lies in their strong N metabolism capabilities in the roots, which allow for more efficient utilization of the limited N sources in the soil. Additionally, pine forests exhibit a strong antioxidant stress response, enabling them to maintain growth in relatively poor and arid environments, demonstrating a strong capacity for survival and adaptation.

## Figures and Tables

**Table 1:** Basic characteristics of meadow plots at different elevations.

Plot ID	Forest Type	Elevation (m)	Longitude (°E)	Latitude (°N)	Mean Vegetation Cover (%)	Mean Diameter at Breast Height (m)	Mean Tree Height (m)	Vegetation Composition
1	Pure forest of *Picea wilsonii*	2328	102°28′12′′	36°56′39″	45	18.93	15.66	The overstory is dominated by *Picea crassifolia*. The understory shrub layer is primarily composed of *Ribes spinosum* (spiny currant), *Lonicera tangutica* (Tangut honeysuckle), and *Rosa acicularis* (prickly rose). The herbaceous layer includes species such as *Fragaria orientalis* (Oriental strawberry), *Dryopteris* spp. (wood ferns), and *Vaccinium vitis-idaea* (lingonberry).
2	Pure forest of *Betula*	2941	102°18′8″	36°54′53″	72	8.21	7.47	The overstory is dominated by *Betula platyphylla* (Asian white birch). The understory shrub layer mainly includes *Myricaria germanica* (tamarisk), *Rhododendron przewalskii* (Przewalski’s rhododendron), and *Rosa sericea* (Silky rose). The herbaceous layer comprises *Fragaria orientalis* (Oriental strawberry), *Dryopteris* spp. (wood ferns), and *Rubus* spp. (raspberries).
3	Pure forest of *Juniperus squamata*	3255	102°16′10″	36°53′36″	60	14.81	5.79	The overstory is dominated by *Juniperus przewalskii* (Qilian juniper). The understory shrub layer mainly includes *Potentilla fruticosa* (shrubby cinquefoil), *Berberis* spp. (barberry), and *Sempervivella* spp. (assumed “Dongxiaqing”—further taxonomic confirmation may be needed). The herbaceous layer consists of *Fragaria orientalis* (Oriental strawberry), *Polygonum viviparum* (alpine bistort), and *Poa pratensis* (Kentucky bluegrass).
4	Pure forest of *Pinus tabuliformis*	2378	102°27′18″	36°57′35″	75	15.36	13.63	The overstory is dominated by *Pinus tabuliformis* (Chinese pine). The understory shrub layer primarily includes *Dasiphora nivea* (silvery cinquefoil). The herbaceous layer is mainly composed of *Fragaria orientalis* (Oriental strawberry) and *Carex* spp. (sedges).
5	Mixed coniferous and broadleaved forest	2512	102°24′51″	36°55′8″	70	22.91	19.26	The overstory consists of *Betula albo-sinensis* (Chinese red birch) and *Picea crassifolia* (Qinghai spruce). The understory shrub layer mainly includes *Lonicera tangutica* (Tangut honeysuckle) and *Rosa sericea* (Silky rose). The herbaceous layer is primarily composed of *Dryopteris* spp. (wood ferns).
6	Mixed broadleaved forest	2559	102°22′39″	36°55′27″	75	25.08	15.62	The overstory is composed of *Betula platyphylla* (white birch) and *Betula albo-sinensis* (Chinese red birch). The understory shrub layer primarily includes *Lonicera tangutica* (Tangut honeysuckle). The herbaceous layer is dominated by *Vicia amoena* (wild vetch), *Saussurea* spp. (saw-wort), and *Thalictrum* spp. (meadow-rue).

## Data Availability

The original contributions presented in this study are included in the article. Further inquiries can be directed to the corresponding authors.

## Abbreviations

The following abbreviations are used in this manuscript:

AK	Available potassium
ALP	Phosphatase
AN	Alkali-hydrolysable nitrogen
AP	Available phosphorus
BD	Bulk density
CAT	Catalase
EC	Electrical conductivity
HS	Pure forest of *Betula*
KKHJ	Mixed broadleaved forest
NCM	Neutral community model
OTU	Operational taxonomic unit
PCR	Polymerase chain reaction
QQ	Pure forest of *Picea wilsonii*
RDA	Redundancy analysis
SOC	Soil organic carbon
SUC	Sucrase
SWC	Soil water content
TC	Total carbon
TK	Total potassium
TN	Total nitrogen
TP	Total phosphorus
URE	Urease
YB	Pure forest of *Juniperus squamata*
YS	Pure forest of *Pinus tabuliformis*
ZKHJ	Mixed coniferous and broadleaved forest
